# Musculoskeletal Biomarkers Response to Exercise in Older Adults

**DOI:** 10.3389/fragi.2022.867137

**Published:** 2022-07-04

**Authors:** Eduardo L. Abreu, Amy Vance, An-Lin Cheng, Marco Brotto

**Affiliations:** ^1^ School of Nursing and Health Studies, University of Missouri-Kansas City, Kansas City, MO, United States; ^2^ University of Missouri Extension, Columbia, MO, United States; ^3^ Department of Biomedical and Health Informatics, School of Medicine, University of Missouri-Kansas City, Kansas City, MO, United States; ^4^ Bone-Muscle Research Center, College of Nursing and Health Innovation, University of Texas at Arlington, Arlington, TX, United States

**Keywords:** exercise, aging, musculoskeletal, biomarker, troponin T, sclerostin, osteocalcin

## Abstract

Exercise is an essential component of any good health style, being particularly important for older adults to counteract the effects of aging, including sarcopenia and osteoporosis, which can result in lower fall probability. Exercise programs for older adults are especially designed for that population. A rigorous evaluation of those programs is necessary to assure most benefit is achieved. Serum biomarkers of proteins intrinsic to musculoskeletal homeostasis could contribute objectively to the assessment of the benefits of exercise. In this work, in addition to the usual physical fitness and balance tests, ELISA assays quantified the serum levels of six proteins and one polysaccharide important for the homeostasis of muscle (troponin T and alpha-actinin), tendon/ligament (tenomodulin), cartilage (cartilage oligomeric matrix protein and hyaluronan) and bone (osteocalcin and sclerostin), before and after 8 weeks of an exercise program tailored to older adults, Stay Strong Stay Healthy, offered at a Community Center and at an Independent Senior Living facility. Statistical significance was determined by non-parametric tests (Wilcoxon Signed Ranks and Mann-Whitney U). Physical fitness and balance improved as expected along with a significant decrease in sclerostin, pointing to less inhibition of bone deposition. However, when considering each type of dwelling separately, older adults always saw a significant decrease of the isoform of troponin T associated with fast-twitch muscles, suggesting that daily levels of physical activity may also have a role in the benefit of older adults from exercise.

## Introduction

Exercise is an essential component of any good health style (Gil-Salcedo et al., 2020; Carapeto and Aguayo-Mazzucato, 2021; Lampek et al., 2021; St Quinton et al., 2021; Townsend et al., 2021) and the benefits of exercising at all age groups (Gil-Salcedo et al., 2020; Clyne and Anding-Rost, 2021; Lampek et al., 2021; St Quinton et al., 2021) and under different medical conditions (Bray et al., 2021; Clyne and Anding-Rost, 2021; Ferrari et al., 2021; Townsend et al., 2021; Gorzelitz et al., 2022) have been extensively reported in the literature. Aging is followed in general by a physiological decline in body functions and increased probability of some diseases (Carapeto and Aguayo-Mazzucato, 2021). Under a societal point of view, as the populations get older, initiatives to reduce the effects of aging on health are particularly important (Gil-Salcedo et al., 2020; Lampek et al., 2021). Sarcopenia and osteoporosis, considered twin diseases in the musculoskeletal (MSK) system with high prevalence in older adults, are associated with worse outcomes, including higher morbidity and mortality, following falls (Huang et al., 2014; Brotto et al., 2016; Laskou et al., 2021). A successful approach to mitigate the effects of these conditions should consider a combination of exercise, particularly resistance training, and good nutritional approach (Giangregorio et al., 2015; Liao et al., 2019; Daly et al., 2020; Kirk et al., 2020).

Although the health benefits from exercise, particularly for older adults, are unquestionable, care must be taken to have regimens appropriate for each age group. In this regard, proper evaluation of the effects of exercise become even more important. Traditionally the evaluation to determine improvements in health after exercise in older adults has been done by functional tests to assess physical performance, questionnaires that evaluate parameters such as cognition, fear of falling, quality of life, among others, and sometimes balance and gait analysis (Gschwind et al., 2013; Zhuang et al., 2014; Bates et al., 2018). All valuable evaluations that provide important information to support the value of exercise in the aged population. However, it would also be relevant, adding to the repertoire of tools used to assess the benefits of exercise and physical activity for older adults, the ability to measure those benefits in a more objective way, for example, with serum biomarkers.

In a previous work (Abreu et al., 2014), groups of older adults undergoing two different types of exercise programs, Stay Strong Stay Healthy (SSSH) and Peer Exercise Program Promotes Independence (PEPPI), designed for that age group, had a decrease in their respective serum levels of skeletal muscle troponin T (sTnT), in addition to gains in physical fitness and muscle strength after both exercise interventions. The protein sTnT is part of the sarcomere, the functional unit of muscles, and should be found in serum only at baseline levels in consequence of physiological muscle turnover. Higher serum sTnT would be associated with muscle waste, and could be a potential marker in sarcopenia, or as a consequence of strenuous exercise (accelerated muscle turnover). A decrease in sTnT levels in older adults would be an indication of mitigated muscle damage.

The concept that a panel of biomarkers can be more advantageous than an individual biomarker has been reinforced by recent works, notably in ovarian cancer detection (Zhu et al., 2011; Muinao et al., 2019) and for early intervention in myocardial infarction (Lakhani et al., 2018). Encouraged by our previous results and this recent understanding on biomarkers panels, we decided to explore other serum proteins (and polysaccharide) that are associated with musculoskeletal tissues (muscle, tendon/ligament, cartilage, bone) as potential biomarkers of musculoskeletal health as related to aging, physical inactivity, and response to exercise. In the next paragraphs, the rationale for those potential biomarkers is detailed.

At the time of that initial study (Abreu et al., 2014), the available commercial ELISA kit did not distinguish between the fast- and slow-troponin T isoforms, respectively predominant in fast- and slow-twitch muscles. In this study, however, both isoforms were evaluated. Additionally another sarcomeric protein, alpha-actinin, was investigated. Alpha-actinin is a structural sarcomeric protein that has an important role in actin-crosslinking in the Z-lines of sarcomeres (Ribeiro et al., 2014). Alpha-actinin is in fact a family of conserved proteins involved in actin crosslinking with other sarcomeric and cytoskeleton proteins. The isoforms alpha-actinin-1 and alpha-actinin-4 are non-sarcomeric and are involved in the crosslink of action and other cytoskeleton proteins, while alpha-actinin-2 and alpha-actinin-3 anchors the actinin filaments to the sarcomere Z-line (Hsu et al., 2018). Ogura and others showed that endurance training led to an increase in alpha-actinin-2 of adult and old rats (Ogura et al., 2011), but no work has assessed changes in alpha-actinin in humans, particularly in older adults, following exercise.

Tenomodulin (Tnmd) is a member of the family type II transmembrane glycoproteins (Qi et al., 2012; Lin et al., 2017). It is a known marker of tendon and ligament differentiation, where it is mostly expressed; though also expressed, but less significantly, in other tissues of the body (Lin et al., 2017). Tnmd is also known for its anti-angiogenic function (Qi et al., 2012; Alexandrov and Naimov, 2016) and genetic polymorphisms have been associated with other metabolic conditions, such as obesity, diabetes, lipid metabolism disfunction (Tolppanen et al., 2008; Qi et al., 2012; Alexandrov and Naimov, 2016). Increased TNMD mRNA was found in damaged tendons (Alexandrov and Naimov, 2016).

Cartilage Oligomeric Matrix Protein (COMP) is a glycoprotein that stabilizes collagen type II and regulates water content in articular cartilage (Jayabalan et al., 2021). COMP has been recognized as a marker of cartilage damage (Kraus et al., 2011; Kraus et al., 2015; Cho and Roh, 2016; Henrotin et al., 2016). Hyaluronan, or hyaluronic acid, (HA) is a ubiquitous extracellular matrix linear polysaccharide, consisting of repeating disaccharides (glucuronic acid and glucosamine); despite its simplicity, in addition to its role in the musculoskeletal system, HA also has an ample array of biological functions in the body (Dicker et al., 2014). As a cartilage damage biomarker, serum HA serum levels were able to discriminate normal from symptomatic osteoarthritis (OA) (Singh et al., 2014) and have been associated with radiographic knee and hip OA (Elliott et al., 2005). Serum levels of HA, as well as COMP, has been shown to be sensitive to early OA in the knee joint (Jiao et al., 2016). It has also been reported that HA was able to predict the incidence of hand OA (Saruga et al., 2021).

Osteocalcin (OCN) is the most abundant non-collagenous bone protein, an osteoblastic hormone that has been correlated with bone formation and number of osteoblasts by several studies and, in addition, regulates many other physiological processes, for example, glucose and energy metabolism (Oldknow et al., 2015; Otani et al., 2015; Wei and Karsenty, 2015; Zeng et al., 2021). Total serum OCN includes both carboxylated (cOCN) or uncarboxylated (ucOCN) forms; cOCN is predominantly located in bone, while ucOCN is related to glucose metabolism (Smith et al., 2021). Sclerostin (SOST) is a small bone protein produced by osteocytes that has an inhibitory effect on osteoblasts, hence preventing bone formation (Lewiecki, 2014). Therefore, SOST inhibition has been considered for the treatment of osteoporosis (Lewiecki, 2014; Rauch and Adachi, 2016).

Aging in place is a concept that is continuously evolving (Vanleerberghe et al., 2017) and its detailed discussion is outside the scope of this paper; in basic terms, it means the ability of older adults to stay at their homes or at least in their communities, as they continue to age (Kim et al., 2017; Vanleerberghe et al., 2017; Lewis and Buffel, 2020). Most older adults prefer to stay in the community instead of moving to a residential (independent or assisted living facility) or nursing home (Peek et al., 2016; Kim et al., 2017; Fritz and Dermody, 2019; Lewis and Buffel, 2020). To allow it to happen, it is important to develop technologies that will improve the safety and viability of this dwelling option (Rantz et al., 2013; Peek et al., 2016; Kim et al., 2017; Fritz and Dermody, 2019). Advantages for older adult to stay at their homes are maintaining their independence and keeping their sense of attachment to the community, not to mention economic savings. However, as a matter of convenience, older adults who are still able to maintain some independence may decide to move into an independent-living community to have access to meal planning, scheduled social activities, medical care, housekeeping, and other services. In those cases, they may eventually leave the facility to dine out, visit friends, go to healthcare appointments. Other times, moving out of their homes into an assisted-living or nursing home may be dictated by the need of more intensive care and supervision. Interestingly, most works reporting exercise interventions for older adults have directed their attention to community-dwelling older adults (Gillespie et al., 2012; Ball et al., 2013; Zhuang et al., 2014; Bates et al., 2018).

The overall hypothesis is that exercise under the defined conditions of this study can alter the serum levels of the investigated, MSK-related, biomarkers. The objective is the determination of biomarkers for a panel that can be used to better understand the relationships between exercise and aging.

## Materials and Methods

### Regulatory Approval

A study protocol was approved, and permission granted for this community-based study by the University of Missouri-Kansas City Institutional Review Board (UMKC IRB #15-454).

### Recruitment and Location

Participants were recruited via flyers and word of mouth at the two different sites where Stay Strong Stay Healthy had been regularly offered free of charge to older adults. The first site was an Independent Senior Living facility in Kansas City, MO (“Independent Living Facility”); while the second one was a community center maintained by the Clay County Senior Services in Smithville, MO (“Community Center”) that provides different activities to older adults living in the community. After being told about the nature of the study, those willing to participate were consented by the co-author responsible for the exercise sessions (AV). The work was conducted first at the Independent Living Facility and afterwards at the Community Center and run, along with the physical and balance evaluations by one of the co-authors (AV), an experienced SSSH trainer who was at the time with University of Missouri (MU) Extension. All participants were asked to answer the “Physical Activity Readiness Questionnaire” (PAR-Q), a short “Find your Start Point” questionnaire (optional), and to present a release form signed by a physician before enrolling in the study.

### Exercise Intervention—Stay Strong Stay Healthy

Stay Strong Stay Healthy (SSSH) was chosen as the exercise intervention for this study since no difference was seen in an initial study between the two exercise programs used at the time, PEPPI and SSSH (Abreu et al., 2014). However, this time we investigated the response to exercise in two different populations according to dwelling, older adults in an independent living or still living at their homes. The 8-week, 1-h, twice-a-week SSSH program consisted of warm-ups and upper and lower body strengthening exercises with and without free weights. More detailed information about SSSH, including a graphical description of the exercise, can be found in the literature (Ball et al., 2013).

### Physical Fitness Tests

Five functional performance tests were used to evaluate changes in physical fitness before and after the 8-week SSSH exercise program: 30-s chair stand, chair sit-and-reach, back scratch, 8-foot up-and-go and grip strength. These tests have been successfully applied to evaluate fitness in older adults and their use have been widely reported in the literature. They are described in detail on previous papers (Ball et al., 2013; Abreu et al., 2014). The tests along with a balance evaluation were performed at the end of the first and last day of the SSSH program.

### Balance Evaluation

Balance evaluation was done differently at both study locations. First, at the Independent Senior Living Facility, the evaluation consisted of an adapted Berg Balance Scale, six selected tasks with scores varying between 0 and four for all tasks, which were sitting to standing, standing with eyes closed, reaching forward with outstretched arm, retrieving object from floor, turning to look behind, standing with one foot in front and standing on one foot. The Berg Balance Scale has been successfully used to evaluate the effects of exercise in older adults (Gleeson et al., 2014; Aartolahti et al., 2020; Sadjapong et al., 2020). Later, at the Community Center, three unassisted standing positions (mountain pose, tandem stand and one-legged stand), which have been previously described in Ball et al. (Ball et al., 2013) were recorded in seconds.

### Biomarkers of Musculoskeletal Health and Disease

The biomarkers were quantified in serum using ELISA assays following the respective manufacturers’ instructions. The following sandwich ELISA kits were used: Fast and slow sTnT, CTX-1 and Tnmd from MyBio Source; COMP, HA and SOST from R&D System; OCN and alpha-actinin-2 from Invitrogen. At the end of the first and last day of SSSH, after the physical fitness and balance evaluations, participants had their blood drawn into serum separator tubes by a skilled nurse and kept in cold until back in the lab. The length of time for blood drawing was consistent among participants, at both locations and at both times, within 15-35 min after exercise. Serum was then separated by manufacturer’s instructions, and aliquoted into eight tubes to be used on the different ELISA assays. Aliquots were stored at -80°C until used and serum samples were thawed only once. For each biomarker, in addition to the measured serum concentrations, we also calculated each subject’s percent change between the two measurements, before and after SSSH.

### Statistical Analysis

Non-parametric statistical tests were used throughout the study since results were not normally distributed. Differences between measurements before and after SSSH for the biomarkers (serum concentration), functional tests and balance scores were evaluated using the Wilcoxon Signed Ranks Matched Paired test. When comparing the biomarkers results by type of dwelling, the Mann-Whitney U tests were applied to the percent changes between before and after SSSH. Statistical values were reported as mean and standard deviation for pre- and post-SSSH measurements; median and interquartile range for the differences between post- and pre-SSSH. For all statistical tests the type 1 error rate was set at *p* < 0.05.

## Results

### Attrition, Attendance, and Demographics

From the initial 21 participants recruited, two dropped from the study for unknown reasons, both from the Independent Living Facility, resulting in a final sample size of 19 participants, ten (10) at the Independent Living Facility and nine (9) at the Community Center, corresponding to an attrition of approximately10%. Attendance for individual subjects varied between 87.5 and 100% for the 16 SSSH classes at the two places. Except for one male subject, all others were females, and ages varied between 65 and 90 years with mean (SD) age of 72.9 (5.6) years.

### Physical Fitness

Participants showed improvements in flexibility and upper/lower body strength based on the statistically significant changes seen after the SSSH exercise program for all functional performance tests. Table 1 shows for each test mean and SD (pre- and post-SSSH), median and interquartile range (IQR), mean percent change (in relation to initial measurement), and *p*-value (Wilcoxon Signed Ranks Matched Paired test).

**TABLE 1:** Results of functional tests before and after the 8-week exercise program Stay Strong Stay Healthy (SSSH). Percent change shown as absolute number.

Physical fitness tests	Pre-SSSH	Post-SSSH	Post-pre	Mean % Change	p
	Mean (SD)	Mean (SD)	Median (IQR)		
30-s chair stand	14.0 (4.3)	18.6 (5.1)	3.5 (5.5)	32.9	0.001
Chair sit-and-reach (in)	2.2 (3.1)	3.4 (3.1)	0.9 (2.4)	69.5	0.024
Back scratch (in)	-7.8 (3.3)	-7.2 (3.4)	1.0 (1.4)	7.7	0.014
8-foot up-and-go (sec)	5.8 (1.7)	4.8 (1.4)	-1.0 (2.0)	17.2	0.004
Grip strength (kg)	23.6 (6.9)	25.4 (7.6)	1.4 (2.3)	7.6	0.001

Pre-SSSH and post-SSSH values were significantly different for all tests (Wilcoxon Signed Ranks Matched Paired test, *p* < 0.05) (SD = standard deviation; IQR = interquartile range).

### Balance

As mentioned in the previous section, two different evaluations for balance were used at the Independent Living facility (adapted Berg Balance Scale) and at the Community Center (standing poses). The reason for these two evaluations is explained in the Discussion section. The results from the Berg test were [mean (SD)] before and after the SSSH classes, respectively, 18.9 (2.8) and 21.9 (1.2), representing a significant (*p* = 0.02) improvement of 15.9%. In relation to the Community Center, results were 24.7 (3.5) and 26.1 (4.9) before and after SSSH, respectively, a non-significant result (*p* = 0.20).

### Biomarkers of Musculoskeletal Health and Disease

**FIGURE 1:**
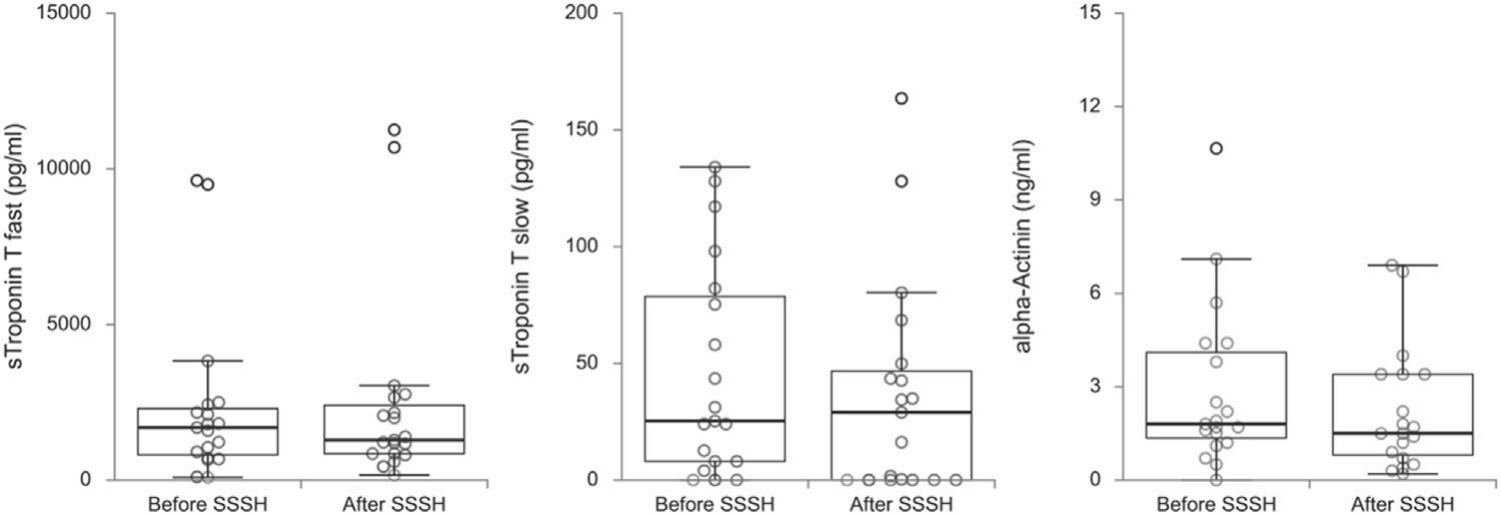
Box plots of muscle-related biomarkers skeletal muscle troponin T, fast and slow, and alpha-Actinin before and after the exercise program SSSH.

**FIGURE 2:**
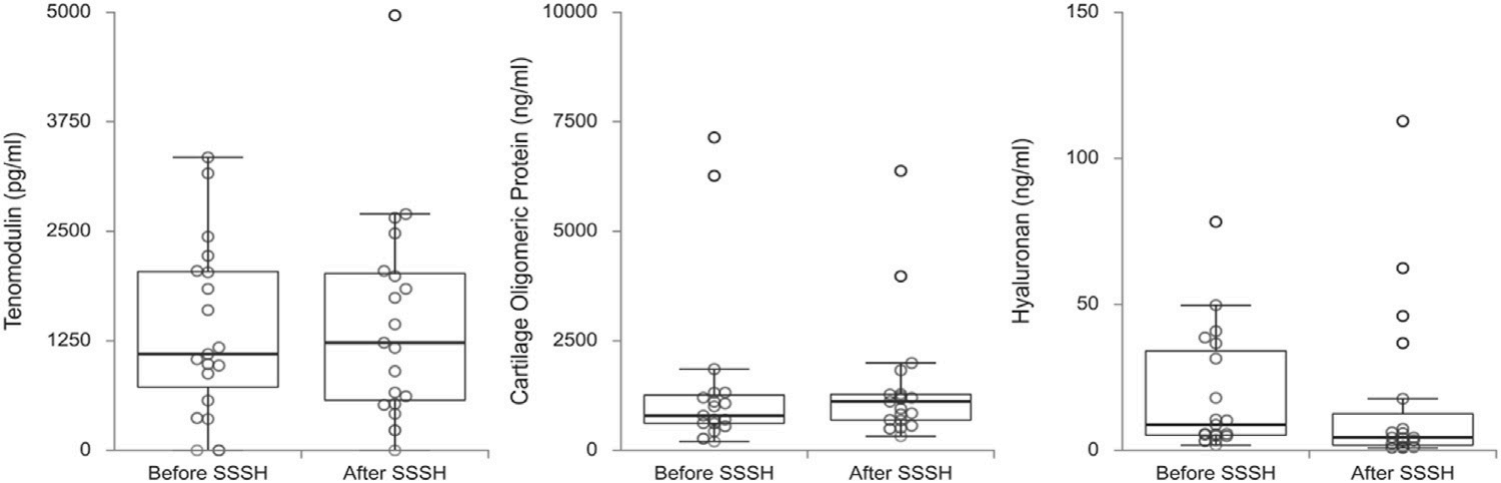
Box plots of tendon/ligament-related (tenomodulin) and cartilage-related (cartilage oligomeric matrix protein and hyaluronan) biomarkers before and after the exercise program SSSH.

**FIGURE 3:**
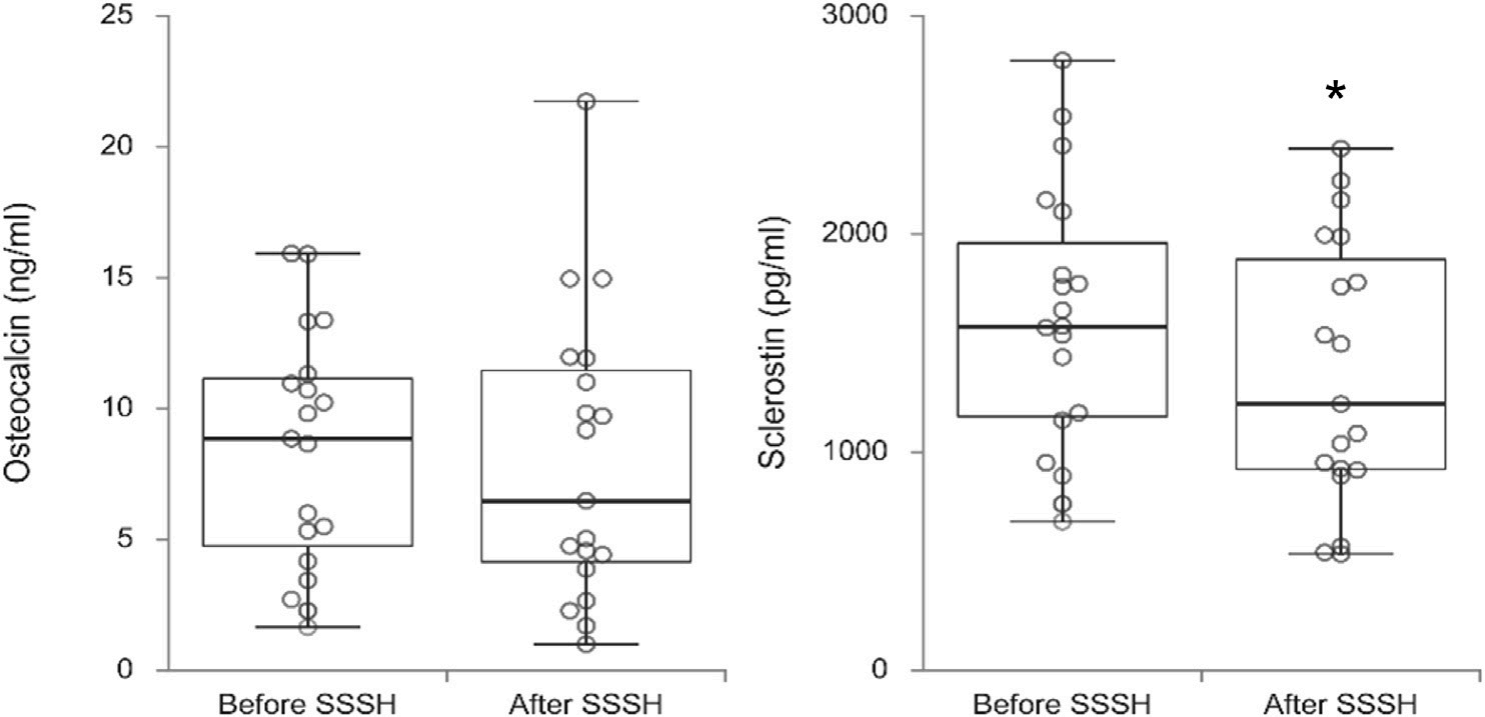
Box plots of bone-related biomarkers osteocalcin and sclerostin before and after the exercise program SSSH. Significance indicated by * (*p* = 0.003)

Comparisons between potential serum biomarkers for muscle, tendon/ligament and cartilage, and bone before and after an 8-week exercise program (Stay Strong Stay Healthy) are shown respectively in Figures 1–3. Table 2 shows for each biomarker mean and SD (pre- and post-SSSH), median and interquartile range (IQR), mean percent change (in relation to initial measurement), and *p*-value (Wilcoxon Signed Ranks Matched Paired test).

**TABLE 2:** Results of serum biomarkers tests before and after the 8-week exercise program Stay Strong Stay Healthy (SSSH). Percent change shown as absolute number.

Biomarkers	Pre-SSSH	Post-SSSH	Post-pre	Mean % Change	p
	Mean (SD)	Mean (SD)	Median (IQR)		
Tenomodulin (pg/ml)	1375.1 (981.5)	1480.4 (1187.5)	-129.6 (1088.0)	68.7	0.948
Αlpha-Actinin (pg/ml)	2865.6 (2641.9)	2225.7 (1981.2)	59.1 (2385.0)	1.5	0.687
Sclerostin (pg/ml)*	1617.4 (600.9)	1369.5 (605.9)	-222.1 (461.0)	15.3	0.003
Hyaluronan (ng/ml)	19.1 (21.0)	16.9 (29.0)	-3.2 (11.1)	40.9	0.117
Cartilage Oligomeric Protein (ng/ml)	1467.9 (1894.5)	1440.1 (1442.7)	31.6 (175.2)	25.9	0.520
sTroponin-T fast (ng/ml)	2.34 (2.70)	2.45 (3.11)	-0.003 (0.87)	4.1	0.658
sTroponin-T slow (pg/ml)	46.0 (46.1)	36.5 (46.4)	0.00 (25.16)	9.5	0.496
Osteocalcin	8.43 (4.53)	8.00 (5.51)	0.30 (1.84)	4.7	0.952

* Indicates pre-SSSH and post-SSSH values significantly different (Wilcoxon Signed Ranks Matched Paired test) (SD = standard deviation; IQR = interquartile range).

For all participants, regardless of dwelling, the difference in mean serum levels between before and after SSSH was statistically significant only for SOST, *p* = 0.003), in this case, a decrease in serum SOST after SSSH in relation to before SSSH. For all other comparisons, *p*-values varied between 0.117 and 0.952.

**FIGURE 4:**
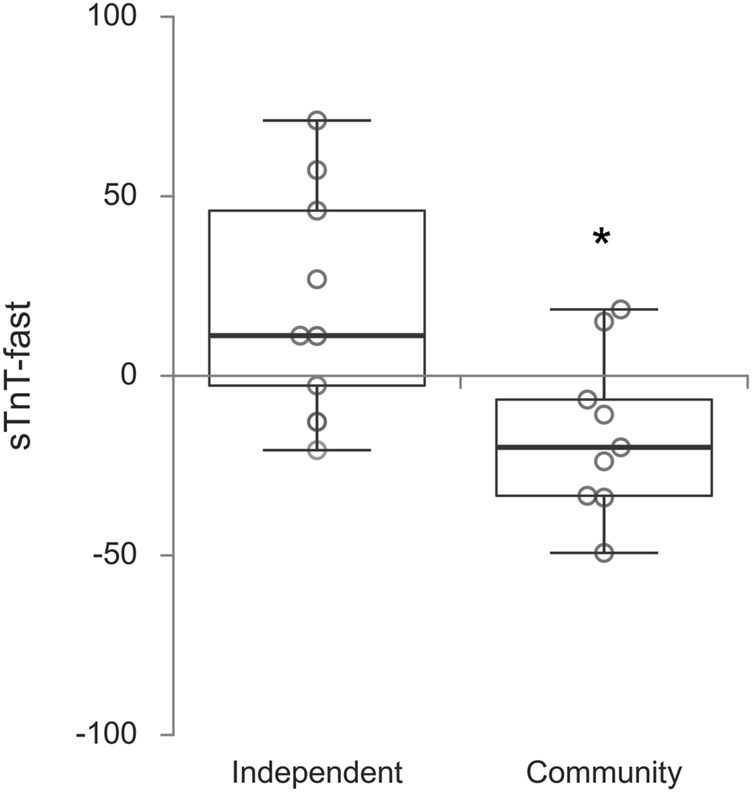
Box plots of percent changes after the exercise program SSSH in skeletal muscle troponin T fast per dwelling. Significance indicated by * (*p* = 0.014)

When reviewing the data, more specifically percent changes, it was noticed that for some biomarkers there was a clear distinction in pattern of percent increase versus decrease (for the paired values) depending on the type of dwelling. Percent changes were then tested for all biomarkers utilizing the Mann-Whitney *U* test, two-tailed. A significant difference for sTnT fast (*p* = 0.014) was found, with mean percent increase of 20.8% for those individuals in the independent living facility [median (IQR) = 19.0 (53.7)] versus a decrease of 16% for those still living in the community [median (IQR) = -19.9 (26.8)]. Furthermore, HA and OCN had close to significance *p*-values (respectively *p* = 0.050 and *p* = 0.060), which warrants further investigation on larger studies. Figure 4 shows percent changes (between pre- and post-SSSH) for sTnT fast for the Independent Living Facility and Community Center groups.

## Discussion

The current study, in addition to the commonly used physical fitness assessment, investigated six serum proteins and one ubiquitous polysaccharide as possible targets for a biomarker panel for the evaluation of the effects of exercise on the musculoskeletal system of older adults.

### Physical Fitness and Balance

All measures of flexibility and upper/lower body strength significantly improved after 8 weeks of exercise intervention (SSSH). Currents results were not only in line with a previous study (Abreu et al., 2014), but also consistent to other works that used functional tests to evaluate on older adults the effects of different exercise programs (Sousa et al., 2014; Zhuang et al., 2014; Furtado et al., 2015). Handgrip strength measurements, when considering age and gender, were compatible with reference values for residents in the US (Wang et al., 2018). They were also in the range but slightly higher in absolute values (and post-exercise percent increase) than in that previous work, despite the current shorter time in this case of the SSSH Program (eight versus 10 weeks). Similar to functional tests, exercise programs in general lead to improvements in grip strength (Labott et al., 2019). Although functional tests and handgrip strength are valid indicators of the benefits of exercise to older adults, they may be less useful to make a distinction between different exercise regimens because in the great of majority of studies there is an improvement in those parameters.

In relation to balance, unfortunately how balance was evaluated needed to be changed. At the Independent Facility, the initial place of this study, the evaluation was done using the adapted Berg Balance Scale. However, during the post-SSSH evaluation one participant fell. The co-author running that part of the study decided to change how balance was evaluated at the Community Center. Although the adapted Berg Balance Scale was able to show improvements in balance following SSSH for the Independent Facility participants, the simpler evaluation at the Community Center did not show any difference. indicating the need of a better way of balance assessment.

### Biomarkers of Musculoskeletal Health and Disease

When analyzing the data, the large variability in the serum biomarkers measurements, with standard deviations (SD) around 50% of the means, was clearly noticeable. Interestingly, this large variability was also seen in similar works in the literature (mentioned later in the Discussion). Possible explanations are the diverse biological processes that can influence the serum levels of those proteins (and polysaccharide) and how much there is still to know about them.

When participants were not divided by type of dwelling, only SOST serum levels were significantly different between pre- and post-SSSH. SOST has been described a “bone formation brake”, being one of the two major inhibitors of the pathway related to bone formation by osteoblasts (Rauch and Adachi, 2016). Therefore, a decrease in mean serum SOST after SSSH suggests less inhibition of bone deposition as part of bone homeostasis. Furthermore, when considering the effect of resistance exercise on muscles, the decline seen in circulating SOST supports further investigation on the role of muscle-bone crosstalk on the beneficial overall impact of exercise on musculoskeletal health.

When the data was analyzed considering the type of dwelling (Independent Living versus Community Center) other significant (or close to significance) differences between pre- and post-SSSH emerged in the Community Center group for TnT fast, HA and OCN. Which introduces an interesting point. Does the daily life level of activity affect the response of older adults to exercise? One possible way of answering this question would be the monitoring of the level of activity of each participant outside the exercise classes by wearable activity trackers. It would also be interesting to consider other types of dwelling, for example, assisted living and nursing homes. It is opportune to mention that studies investigating the response of older adults to exercise have focused predominantly on community-dwelling populations.

This study results were comparable to previously reported findings for COMP (Wakitani et al., 2007; Das Gupta et al., 2017; Jayabalan et al., 2021); HA (Montazeri et al., 2005; Singh et al., 2014); OCN (Khwanchuea and Punsawad, 2021; Nobrega da Silva et al., 2021); and SOST (Bhattoa et al., 2013).

In relation to the other proteins (sTnT, alpha-actinin and Tnmd), only one study has investigated previously serum levels sTnT (Abreu et al., 2014), while there are only genetic works on the physiological or pathological roles in humans of alpha-actinin and Tnmd.

The current work built upon previous work that found a decrease in serum skeletal muscle troponin T (sTnT), along with improvements in physical performance and grip strength, in older adults after two similar but distinct 10-week exercise programs, “Peer Exercise Program Promotes Independence (PEPPI)” and “Stay Strong Stay Healthy (SSSH)”, both designed having in mind the needs and limitations of older adults (Abreu et al., 2014). No difference in outcomes was found between PEPPI and SSSH, despite their slight differences (Abreu et al., 2014) so for this study the only exercise intervention was SSSH, this time at a shorter time (8 weeks) according to MU Extension current directives. Also, that initial study focused more on community-dwelling older adults and no special attention was paid to other types of dwelling and their intrinsic differences in terms of everyday activity level. Approximately half participants in this study were community-dwelling older adults, while the other half lived in an independent-living facility. Interestingly, sTnT serum concentration results from that previous work are more in line with sTnT slow in the current study.

**FIGURE 5:**
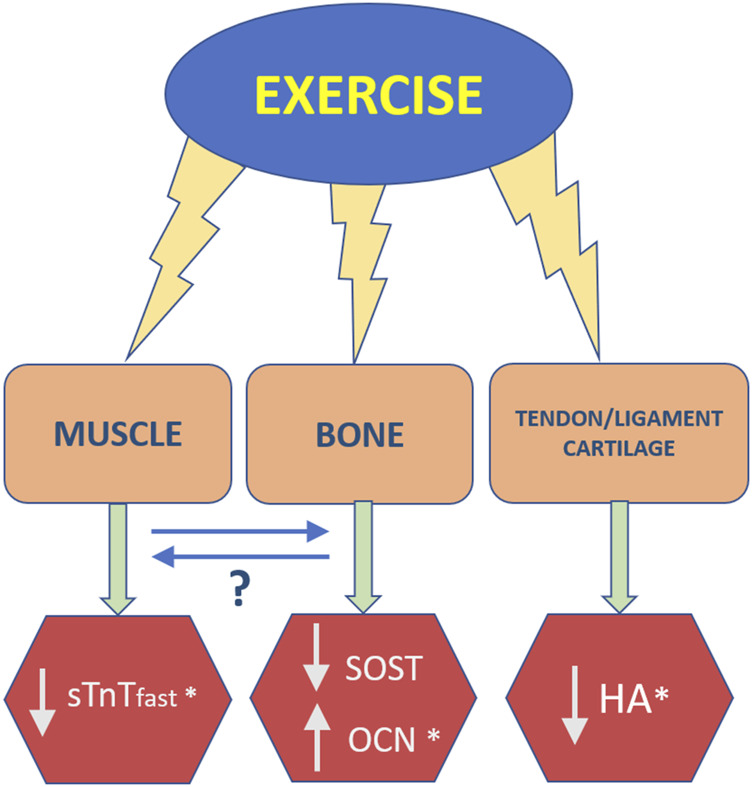
Main effects of exercise on biomarkers associated with tissues of the musculoskeletal system, also showing potential interrelationship or crosstalk between muscle and bone.

In Figure 5, the most important results are summarized graphically, questioning the role of a crosstalk between muscle-bone in the overall response of the musculoskeletal system of older adults to exercise. Interesting points to note are that slow twitch sTnT, more associated with postural muscles, did not seem to be affected by SSSH, despite improvements in balance; the potential role of HA as biomarker for the effects of exercise in older adults, despite its widespread importance in the body; and that SOST and OCN moved in opposite directions of change, consistent with their respective anti-anabolic anabolic roles in bone homeostasis. Also, both studies with similar values for OCN intriguingly investigated overweight adolescents, which may be explained by the role of OCN, on its uncarboxylated form, in energy metabolism (Nobrega da Silva et al., 2021).

### Limitations and Strengths

The main limitations of this work are its sample size and lack of diversity in the population studied, which limit the generalization of the results. Also, balance evaluation was limited and incomplete. On the other hand, this current work results, despite the relatively small sample size and large standard deviations, are encouraging towards a larger study on a more diverse population and include a more rigorous balance evaluation, as previously done (King et al., 2016; King et al., 2019).

## Conclusion

Despite constraints that prevented a larger study, this work raised some interesting matters. First, the significant decline in serum sclerostin levels after 8 weeks of exercise; if it resulted only from the effect of exercise on bones or if muscles were also involved in that outcome. Second, the possible role of daily activity level on the older adults’ response to exercise. Third, the prospect that a larger study could confirm other proteins (and hyaluronic acid) as biomarkers to the body’s response to exercise. Fourth, a more rigorous balance evaluation, probably using proper software and hardware, is necessary. These are desirable objectives for future larger studies.

## Data Availability

The raw data supporting the conclusion of this article will be made available by the authors, without undue reservation.
